# Comparative Assessment of Diet Quality and Adherence to a Structured Nutrition and Exercise Intervention Compared with Usual Care in Pregnancy in a Randomized Trial

**DOI:** 10.1016/j.cdnut.2023.100097

**Published:** 2023-05-13

**Authors:** Kendra Dempsey, Michelle F. Mottola, Stephanie A. Atkinson

**Affiliations:** 1 Department of Pediatrics, McMaster University, Hamilton, Ontario, Canada; 2 School of Kinesiology, The University of Western Ontario, London, Ontario, Canada

**Keywords:** adherence, pregnancy, nutrition, exercise, randomized controlled trial, lifestyle intervention, compliance, protein, dietary recommendations, behavior change

## Abstract

**Background:**

In trials testing the efficacy of diet and exercise modifications during pregnancy on health outcomes, assessment of participant adherence to interventions of diet and exercise is rarely reported, with few standard methods existing to measure adherence.

**Objective:**

We aimed to assess the maternal diet quality and create an algorithm to evaluate adherence to an intervention of high protein/dairy nutrition and walking exercise from early pregnancy to birth.

**Methods:**

In Be Healthy in Pregnancy randomized trial (NCT01693510), diet quality was measured using scores from an adapted PrimeScreen food frequency questionnaire, nutrient intake assessed by 3-day diet records, and physical activity using accelerometry at 14–17 (early), 26–28 (middle), and 36–38 (late) weeks’ gestation. A novel adherence score was derived by combining data for compliance with prescribed protein and energy intakes and daily step counts in the intervention group. Between-group diet quality scores and changes in adherence scores in the intervention group across pregnancy were analyzed using generalized estimating equations adjusted for prepregnancy body mass index and study site.

**Results:**

Diet scores were similar for intervention (*n* = 55) and control (*n* = 56) groups at baseline but only the intervention group significantly improved and maintained their scores from early to middle (18.7 ± 7.6 vs. 22.9 ± 6.1; *P* < 0.001) and late (22.5 ± 6.9; *P* < 0.008) pregnancy. Protein intake was significantly (*P* < 0.001) higher but energy intakes were similar in the intervention group compared with those in the control group. Adherence scores for the intervention increased significantly (*P* < 0.01) from early (1.52 ± 0.70) to midpregnancy (1.89 ± 0.82) but declined from midpregnancy to late (1.55 ± 0.78; *P* < 0.0005) pregnancy primarily owing to lower step counts.

**Conclusions:**

Adherence to an intervention may decline toward the end of pregnancy, particularly in maintaining physical activity. Creation of adherence scores is a feasible approach to measure combined intervention compliance for diet and physical activity and may increase transparency in interpreting results of randomized trials in pregnancy.

This trial was registered at clinicaltrials.gov as NCT01689961 (https://clinicaltrials.gov/ct2/show/NCT01689961?cond=NCT01689961&rank=1; registered on 21 September 2012).

## Introduction

Entering pregnancy overweight and excess or inadequate gestational weight gain (GWG) has been associated with the likelihood of adverse pregnancy-related outcomes for mothers and their offspring, such as hypertension, preeclampsia, gestational diabetes mellitus, postgravid weight retention, macrosomia, higher incidence of birth trauma, and future health implications such as metabolic disorders and obesity [[1], [2], [3], [4], [5]]. Appropriate GWG, as outlined by guidelines from the Institute of Medicine (IOM) [6], is defined in relation to prepregnancy BMI (pBMI). IOM recommendations for total GWG range from 11.5 to 16 kg for the category of normal BMI, 7 to 11.5 kg for overweight BMI, and 5 to 9 kg for the obese BMI [6]. Because an estimated 50%–65% of Canadian females have a GWG in excess of IOM recommendations [7], a critical need exists for developing clinical approaches to combat pregnancy-related excess GWG [8]. Published systematic reviews or meta-analysis that investigated specific lifestyle interventions, of which many were targeted to only overweight and obese females, are inconsistent in their findings on the effect on GWG and maternal and infant health outcomes. A meta-analysis of 117 randomized controlled trials (RCTs) (*N* = 34,546 persons) showed variable but consistent reductions in GWG with interventions of structured diet, exercise, or a combination but only a positive effect of diet alone on infant outcomes [9]. A reduction in relative risk of excessive GWG by 20% but no effect on infant outcomes such as macrosomia was reported in an updated Cochrane review [10].

A major limitation of many published RCTs of lifestyle interventions in pregnancy is the lack of accountability of detailed objective measures of the nutrient profile of diets consumed and physical activity output and participant adherence to the intervention. A pregnancy intervention based on dietary counseling to combat excessive GWG reported no difference in treatment groups, yet simultaneously reported poor presence at counseling sessions but without quantitative measures of dietary intake [11]. Similarly, in a Dutch study that found no intervention effect of promoting biweekly exercise class attendance alongside self-directed physical activity in an overweight pregnant population, low exercise class attendance was reported but without objective measures of physical activity such as step counts or daily energy expenditure [12]. Currently, no standard method exists to quantitatively report intervention adherence, and thus, approaches to measuring adherence are varied. Two RCTs targeting GWG have successfully implemented adherence measures [13,14]; however, such tools were created to correspond to the specific outputs of their respective interventions, and neither developed an algorithm to capture adherence across time. This presents a need to develop methods to best measure intervention adherence.

The first objective of this study was to compare dietary practices and nutrient intakes between intervention and control groups enrolled in a nutrition and exercise lifestyle intervention in pregnancy, using diet scores as an indicator of healthy dietary practices and nutrient intakes compared with recommendations. The second was to create an algorithm to describe adherence to both the nutrition and exercise components of the intervention at different times across pregnancy, to determine whether adherence changed throughout pregnancy. We hypothesized that intervention compared with control participants would have higher healthy diet scores and higher protein intakes maintained across pregnancy. Second, we hypothesized that intervention participants would maintain their adherence to the prescribed nutrition and exercise intervention from early to late pregnancy, as captured by the adherence algorithm created.

## Materials and Methods

### Study design

Data were collected in the Be Healthy in Pregnancy (BHIP) study, a 2-armed, 2-site prospective RCT (NCT01689961; registered on 21 September 2012; https://clinicaltrials.gov/ct2/show/NCT01689961), as previously described in detail [15,16]. The primary objective of the BHIP RCT on which the trial sample size was based was to increase the proportion of females with GWG within the guidelines outlined by the IOM [6]. The intervention comprised an individualized and monitored nutrition and exercise lifestyle regimen that began in early pregnancy (12–17 weeks’ gestation), with an emphasis on consumption of a high-protein, low-fat dairy diet and exercise with a goal of 10,000 steps per day, meeting current guidelines in pregnancy [15]. The BHIP study operated at McMaster University (Hamilton, Ontario, Canada), and Western University (London, Ontario, Canada) between January 2013 and April 2018 for recruitment with follow-up to February 2019. Throughout the study, the intervention protocol and outcome measures remained similar, following standard operating procedures established at the start of the study. The type and brand names of the dairy products provided to the intervention group remained consistent throughout. The study was conducted according to the guidelines of the Declaration of Helsinki and approved by the research ethics boards of Hamilton Health Sciences and McMaster University, Hamilton (REB Project # 12-469, 2012); Western University, London (HSREB 103272, 2013); and Joseph Brant Hospital, Burlington (JBH 000-018-14, 2013), in Ontario, Canada.

In brief, recruitment targeted healthy pregnant females from all pBMI categories except those with extreme obesity (>40 kg/m^2^), who were identified from community health care clinics in Hamilton, Burlington, and London (Ontario, Canada) between 12 and 17 weeks of gestation from January 2013 to April 2018 as detailed in the study design article [15]. Other exclusion criteria included the following: non-English speaking, known contraindications to exercise, severe gastrointestinal diseases, intolerance/dislike of dairy foods, existing prediabetes, smokers, and depression score of >12 on Edinburgh validated scale [15]. At the first contact and the baseline visit, eligibility criteria were screened [15]. Allocation to treatment group by block randomization stratified by study site and pBMI category used by IOM [1] was performed at the second visit to the study center [15]. Regardless of the treatment group, all participants continued to receive their chosen standard prenatal care and were counseled at the baseline visit by the study nutritionist on Health Canada’s nutrition recommendations for pregnancy [17]; they were also provided Canada’s Food Guide alongside the Pregnancy Weight Gain Calculator [18]. Intervention participants were counseled during biweekly visits (a total of 11–12 visits) with a study nutritionist who provided an individualized diet tailored to meet each participant’s calculated estimated energy requirement (EER) [19] with an emphasis on high protein, providing 25% protein energy with ∼50% of total protein intake derived from dairy foods (equal to 4–6 servings of reduced-fat milk, plain yogurt, or cottage cheese) to achieve a consistent proten-to-energy ratio among participants. At the biweekly visits, participants were provided with reduced-fat dairy foods (milk, plain yogurt, or cottage cheese) according to their preferences as detailed previously [15]. Intervention participants were additionally counseled in a controlled walking-based exercise program, with a goal of 10,000 steps daily. If a participant missed a biweekly visit to the study center, the research assistant conducted a phone interview to provide counseling and collect self-report data. The control group received usual care provided by their primary care provider and returned to the study site only for outcome measurements at 26–28 and 36–38 weeks of gestation. As a retention strategy, control participants were invited to join a focus group in the second trimester led by a midwife, with topics including pain relief options during labor and breastfeeding techniques.

### Measures of participant nutrient intake and diet quality

At 12–17, 26–28, and 36–38 weeks’ gestation, all participants included in the analysis for this study completed 3-day diet records (3DDRs) over 2 weekdays and 1 weekend day that included both foods and supplements that were analyzed using Nutritionist Pro diet analysis software (version 5.2; Axxya Systems) and the Canadian Nutrient File (version 2015) to obtain daily intake of nutrients. At the same time points, a diet quality score was computed for all participants using a FFQ adapted for the BHIP study from a validated tool called PrimeScreen that had been used to assess dietary quality in nonpregnant groups [20]. The PrimeScreen FFQ comprised 25 questions that capture the average frequency of consumption of various food items. Simultaneously with the 3DDRs, step counts and energy expenditure were measured using the SenseWear Armband tri-axis accelerometer (Model MF-SW; BodyMedia).

### Measures of intervention adherence for the creation of a scoring algorithm

The adherence score was created using the following 4 specific goals of the BHIP intervention: maintaining an individually prescribed energy intake; sourcing a minimum 25% of daily energy intake from protein; ensuring 50% of protein intake came from low-fat dairy products; and achieving a 10,000 daily step count*.* Data collected on intakes of energy, protein, and dairy protein and step count were used to assign participants an adherence score ranging from 0 to 4, with higher scores corresponding to a greater adherence to the BHIP intervention (Table 1). For each of the 3 dietary components of the adherence score (energy intake, protein intake, and dairy protein intake), the score was binary as failing to meet or exceeding their prescribed dietary goals had the potential to contribute to an inappropriate GWG. Participants were considered adherent and received the full score for each component if they were within 1 standard deviation of their individual target, to allow for some flexibility in achieving dietary goals (i.e., a participant with an energy intake goal of 2000 kcal who consumed 2030 kcal would not be penalized in the scoring system for such a small discrepancy).

**TABLE 1:** Summary of adherence score criteria used for the Be Healthy in Pregnancy study intervention

Criteria	Category	Assessment/score attribution	Possible score ranges^1^
Are participants meeting their individualized EER?	Binary	Score 1: If a participant’s EI is within 1 population-derived SD of their individualized EER, they are adherent. Score 0: If their energy intake is not within 1 population-derived SD of their EER, they are not adherent	0 (nonadherent) or 1 (adherent)
Are participants consuming a high protein (25% of kcal sourced from protein) daily diet?	Binary	Score 1: If a participant’s protein intake is within 1 population-derived SD of 25% of their energy intake, they are adherent. Score 0: If their energy intake is not within 1 population-derived SD of 25% of their energy intake, they are not adherent	0 (nonadherent) or 1 (adherent)
Are participants sourcing 50% of their protein from dairy sources?	Binary	Score 1: If a participant’s protein intake is within 1 population-derived SD of being 50% sourced from dairy foods, they are adherent. Score 0: If a participant’s protein intake is not within 1 population-derived SD of being 50% sourced from dairy foods, they are not adherent	0 (nonadherent) or 1 (adherent)
Are participants walking 10,000 daily steps?	Continuous	Each participant’s daily step count is divided by 10,000 steps, and the fraction value is the point awarded. This is partial adherence. Participants who walk ≥10,000 steps will be considered fully adherent	Score can range anywhere between 0 (no steps, fully nonadherent) and 1 (≥10,000 steps, fully adherent). Scores in between account for partial adherence

Abbreviations: EER, estimated energy requirement; EI, energy intake

1 The range for possible total score is 0–4.

The exercise component of the adherence score (10,000 daily step count) was made a continuous score as any movement achieved by participants was an added benefit in working toward an optimal GWG. However, the more walking completed, the greater the overall health benefit, hence the increasing score.

The decision to equally weight each component score came from a review of the limited studies in pregnancy, which had created their own study-specific adherence scores [13,14]. Both studies placed equal weight on their component scores. The study by Nagpal et al. [14] was a diet and exercise intervention, similar to the BHIP study with multiple goals within the intervention, and weighted each of their 6 intervention components equally within the score. Table 1 outlines the application of this adherence score.

The adherence score was used across pregnancy, corresponding with the 3 study visits completed by intervention participants at 12–17, 26–28, and 36–38 weeks’ gestation. This revealed how diet and physical activity behaviors changed from before intervention onset (12–17 weeks’ gestation) to after intervention implementation (26–28 and 36–38 weeks’ gestation).

### Statistical analysis

A complete case analysis was conducted for participants for whom data were available for all PrimeScreen FFQ, the 3DDR, and accelerometry across pregnancy (12–17, 26–28, and 36–38 weeks’ gestation). A statistical analysis was conducted using IBM SPSS Statistics for Mac, version 26.0 (IBM). All analyses were adjusted for pBMI and study site (McMaster vs. Western) because these were stratification variables for randomization. Descriptive statistics included the means ± SDs of normally distributed continuous data, medians (Q1, Q3) for nonnormally distributed continuous data, and n (%) for categorical data. All continuous data were checked for normality using the Shapiro-Wilks test before the analysis. Linear regression adjusted for maternal pBMI and study site was used to compare dietary intakes between the treatment groups. PrimeScreen diet scores as an indicator of healthy dietary practices, average daily step counts across pregnancy (12–17, 26–28, and 36–38 weeks’ gestation), and adherence scores were analyzed using generalized estimating equations, adjusted for maternal pBMI and study site, assuming autoregressive [AR(1)] correlation structure to consider correlated repeated outcome data within a participant over time. A normal model with an identity link function was used in the generalized estimating equation analysis.

## Results

### Participant characteristics

The CONSORT recruitment figure for recruitment, randomization, and follow-up of the allocation groups analyzed for the primary outcome of GWG was published previously [16]. Retention to the end of pregnancy was 84% for the intervention group and 86% for the control group [16]. For the analysis of the diet quality and adherence to intervention, 111 (55%; 55 intervention participants, 56 control participants) of the 241 participants who were randomly assigned in the BHIP study, had complete data from the PrimeScreen FFQ, the 3DDR, and accelerometry across pregnancy (12–17, 26–28, and 36–38 weeks’ gestation). Most of the participants presented with similar sociodemographic characteristics such as ethnicity (most were Caucasian), being married or partnered, and with a household income of >$75,000 (Supplementary Table 1). The demographic characteristics of participants for the analysis reported in this study (Supplementary Table 1) were similar to the full sample size of participants analyzed for the primary outcome of GWG [16].

### Diet quality between the treatment groups

PrimeScreen diet scores were comparable between intervention and control participants at baseline (12–17 weeks’ gestation) (Table 2). Diet quality scores increased significantly in intervention participants from baseline to 26–28 weeks’ gestation (*P* < 0.001) and to 36–38 weeks’ gestation (*P* < 0.008) and were maintained between 26–28 and 36–38 weeks’ gestation (Table 2). However, in the control group, participant diet scores remained constant across pregnancy (Table 2). PrimeScreen diet scores are measured on a continuous score, with higher scores indicating increasingly healthy general dietary practices. Although the use of nutrient supplements was not included in the diet quality score, >80% of participants reported taking a standard prenatal multinutrient supplement, so supplemental nutrition was an unlikely modifying factor for diet quality.

**TABLE 2:** PrimeScreen diet quality scores for intervention and control groups at 3 gestational stages of pregnancy

Gestational stage (wk)	Intervention (*n* = 55)^1^	Control (*n* = 56)^2^
12–17^3^	18.7 ± 7.6^a^	17.7 ± 8.7
26–28	22.9 ± 6.1^b^	18.7 ± 7.7
36–38	22.5 ± 6.9^c^	18.7 ± 8.5

Values are mean ± SD.

1 Intervention: a vs. b, *P* < 0.001; a vs. c, *P* < 0.008; as analyzed using generalized estimating equations adjusted for maternal prepregnancy body mass index and study site.

2 Control: no differences across time points as analyzed by generalized estimating equations adjusted for maternal prepregnancy body mass index and study site.

3 Measures taken before intervention initiated.

### Nutrient intake between the treatment groups

At baseline, intakes of key macronutrients were similar between treatment groups (Table 3). Energy intakes remained similar across pregnancy and between treatment groups. As designed, protein intake increased at middle and late pregnancy in the intervention but not in the control group, providing a higher percentage of protein energy (Table 3). Compared with recommended intakes in pregnancy [19], the mean intakes of protein were within or exceeded the recommended estimated average requirement for protein (0.66 g/kg in the first half of pregnancy and 0.88 g/kg in the last half of pregnancy) in most individuals in both treatment groups. Protein, fat, and carbohydrate intake as %energy were, on average, within the recommended Acceptable Macronutrient Distribution Range of the Dietary Reference Intakes [19], as noted in Table 3.

**TABLE 3:** Comparison of mean intakes of energy and macronutrients based on 3-day food intake records at 12–17, 26–28, and 36–38 weeks’ gestation in the intervention and control groups and %energy of each macronutrient compared with the Acceptable Macronutrient Distribution Range of the Dietary Reference Intakes (19)

Outcome	Intervention (*n* = 55)	Control (*n* = 56)	*P*^1^	Intervention (%energy)	Control (%energy)	DRI AMDR, % (range)
12–17 weeks’ gestation						
Energy intake(kcal/d)	2139 ± 516	2198 ± 569	0.571	—	—	—
Fat intake (g/d)	79.7 ± 23.5	82.5 ± 24.1	0.535	33.6 ± 6.0	33.8 ± 5.1	20–35
Carbohydrate intake (g/d)	273.7 ± 69.1	285.0 ± 89.3	0.458	51.8 ± 8.7	51.8 ± 6.6	45–65
Protein intake (g/d)	83.6 ± 24.9	88.7 ± 24.7	0.277	15.7 ± 3.2	16.3 ± 3.0	10–35
26–28 weeks’ gestation						
Energy intake(kcal/d)	2199 ± 448	2157 ± 443	0.614			
Fat intake (g/d)	81.7 ± 26.2	83.7 ± 22.4	0.673	33.0 ± 6.1	34.8 ± 5.9	20–35
Carbohydrate intake (g/d)	268.6 ± 56.5	275.3 ± 67.0	0.568	49.2 ± 5.8	51.1 ± 6.3	45–65
Protein intake (g/d)	105.2 ± 27.8	85.5 ± 20.8	<0.001	19.3 ± 4.4	16.0 ± 3.0	10–35
36–38 weeks’ gestation						
Energy intake(kcal/d)	2284 ± 614	2161 ± 465	0.236			
Fat intake (g/d)	80.3 ± 31.9	83.8 ± 25.3	0.516	31.1 ± 6.7	34.6 ± 5.5	20–35
Carbohydrate intake (g/d)	295.0 ± 84.8	271.9 ± 62.1	0.104	51.9 ± 6.1	50.7 ± 7.2	45–65
Protein intake (g/d)	104.1 ± 27.7	87.9 ± 24.0	0.001	18.5 ± 3.4	16.4 ± 3.4	10–35

Values are mean ± SD unless specified.

Abbreviations: AMDR, Acceptable Macronutrient Distribution Range; DRI, Dietary Reference Intakes

1 Differences in nutrient intakes between treatment groups before and after randomization to treatment were analyzed by linear regression adjusted for maternal prepregnancy body mass index and study site.

Despite prescribed energy intakes that were individualized for each intervention participant as per Dietary Reference Intakes recommendations [19], >50% of intervention participants recorded an energy intake below their prescribed EER [19] after the onset of the intervention at 26–28 and 36–38 weeks’ gestation (Table 4). Only approximately one-quarter of the participants met and did not exceed their individualized prescribed EER at 26–28 and 36–38 weeks’ gestation (Table 4). This contrasted with energy intakes observed in early pregnancy when 40% of participants met their predicted EER (Table 4).

**TABLE 4:** The number and proportion of intervention participants (*n* = 55) who were below, achieved, or exceeded their prescribed estimated energy requirements at 12–17, 26–28, and 36–38 weeks’ gestation

Gestational stage (wk)	Below EER, n (%)	Within EER, n (%)	Exceeded EER, n (%)
12–17	16 (29.1)	22 (40.0)	17 (30.9)
26–28	30 (54.6)	14 (25.5)	11 (20.0)
36–38	31 (56.4)	11 (20.0)	13 (23.6)

Participants were considered to have achieved their prescribed EERs if their actual intake was within 1 SD of their EER (19).

Abbreviation: EER, estimated energy requirement

### Measures of prescribed physical activity output

**FIGURE 1:**
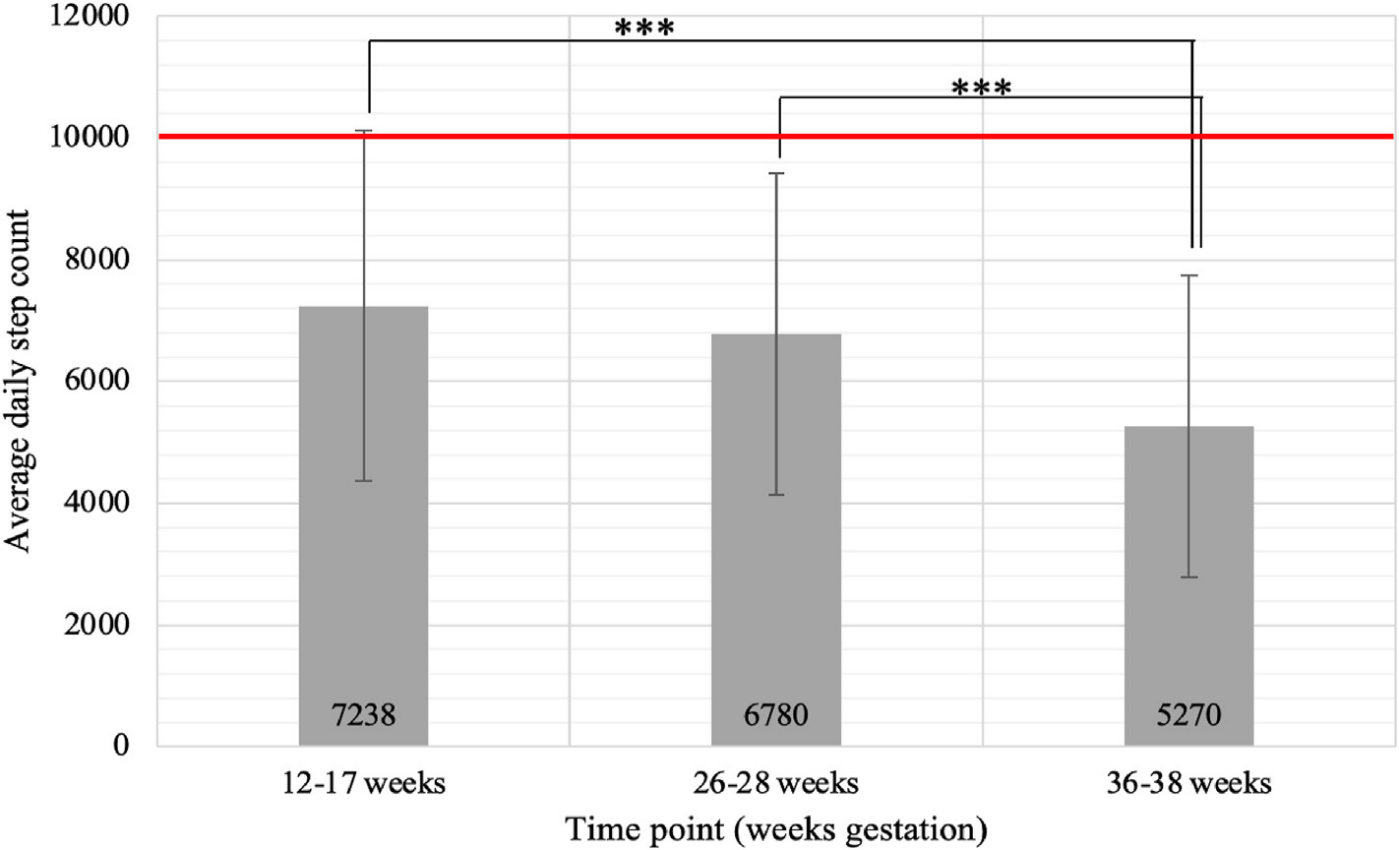
Mean daily step count for intervention participants (*n* = 55) before (12–17 weeks) and after (26–28 and 36–38 weeks) implementation of the intervention analyzed by generalized estimating equations adjusted for prepregnancy body mass index and study site. Error bars are standard deviation; line at 10,000 steps represents prescribed daily step count. ∗∗∗Differences between gestational stage time points, *P* < 0.0005.

Mean daily step counts for intervention participants before and after randomization to the intervention are presented in Figure 1. Mean daily step counts continued to decrease through to the end of pregnancy. Although 14.5% of the intervention participants achieved 10,000 average daily steps at 12–17 weeks’ gestation, this proportion decreased to 9.1% and 5.5% at 26–28 and 36–38 weeks’ gestation, respectively. By comparison with the step counts achieved in the intervention group (Figure 1), daily step counts for the control group were 6527 ± 2845 (mean ± SD) at 26–28 weeks and 5491 ± 2300 at 36–38 weeks’ gestation. Reported activities such as swimming occurred in a small number of intervention (*n* = 8) and control (*n* = 7) participants but only averaged 3–4 min/d during the accelerometry measurement days. Work status was also similar with 90% of the participants in both treatment groups, reporting engagement in full-time work and <1% involved in full-time childcare.

### Creation and application of novel adherence algorithm

**FIGURE 2:**
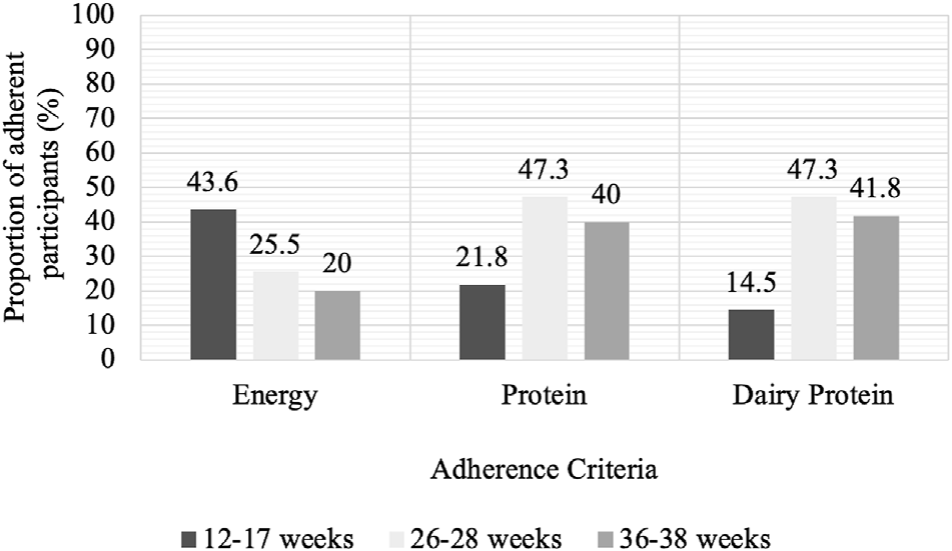
Proportion of intervention participants (*n* = 55) considered adherent to the dietary components of energy, total dietary protein, and dairy protein before (12–17 weeks) and after (26–28 and 36–38 weeks) implementation of the intervention, as defined by the adherence algorithm. Values displayed are percentage of participants.

The proportion of participants who were adherent to the dietary components of the intervention, namely, energy, total dietary protein, and dairy protein intake, are summarized in Figure 2. As pregnancy progressed, fewer participants continued to meet their prescribed EERs. A higher proportion of total dietary protein and dairy protein compared with baseline was achieved after implementation of the intervention and maintained to 36–38 weeks (Figure 2).

The mean adherence scores increased significantly from baseline to 26–28 weeks’ gestation (1.52 ± 0.70 vs. 1.89 ± 0.82; *P* = 0.01), indicating that the prescribed intervention was being implemented (Table 5). However, adherence scores decreased significantly from 26–28 to 36–38 weeks’ gestation (1.89 ± 0.82 vs. 1.55 ± 0.78; *P* < 0.0005), indicating that the adherence to the prescribed intervention was not being maintained at the midpregnancy level (Table 5).

**TABLE 5:** Comparison of adherence scores in the intervention group (*n* = 55) across 3 gestational stages of pregnancy

Parameter	β	95% CI	*P*^1^
Adherence score: 12–17 vs. 26–28 wk	0.35	0.08, 0.63	0.01
Adherence score: 12–17 vs. 36–38 wk	0.02	−0.23, 0.27	0.87
Adherence score: 26–28 vs. 36–38 wk	10.33	−0.51, −0.16	<0.0005
pBMI	0.009	−0.03, 0.05	0.68
Study site	0.02	−0.45, 0.50	0.93

1 Adherence scores analyzed using generalized estimating equations assuming autoregressive [AR(1)] correlation structure, with a normal model with an identity link function and adjusted for prepregnancy body mass index and study site.

Study site and maternal pBMI were not found to affect adherence scores across pregnancy (Table 5). Considering the adherence pattern for dietary components noted in Figure 2 and step count responses noted in Figure 1, the decrease in overall adherence scores toward the end of pregnancy seems to be primarily attributed to the decrease in average daily step count and failure to achieve energy intake requirements for a large proportion of participants through to the end of pregnancy.

## Discussion

In the BHIP randomized trial, the use of diet quality scores to measure change in dietary practices after randomization to the intervention compared with usual care was successful. The intervention participants recorded a higher diet quality PrimeScreen score compared with their habitual self-selected diet at entry to the study, and this was maintained through to the end of pregnancy. The goals for total dietary protein, dairy protein, and energy intake were 100% achieved in close to half of the participants. One can infer that the structured and individualized dietary advice with biweekly counseling from the study nutritionist was effective in improving general dietary practices in the intervention group. This tool also demonstrated that control participants did not change their dietary practices throughout pregnancy. The lower dietary scores in the control group are not necessarily indicative of unhealthy dietary practices but that the dietary practices of the intervention group were healthier by comparison. Although more females in pregnancy report the desire to modify their diets to benefit their offspring compared with nonpregnant populations, many females lack the necessary skills, knowledge, or support to effectively make such changes [[21], [22], [23], [24]]. The frequency of knowledge sharing and guided support from the study nutritionist, in addition to the provision of low-fat dairy foods, may have been extra motivating factors for females enrolled in the intervention group to improve their dietary practices compared with that in the control group.

The goal of 10,000 steps was achieved by <10% of intervention participants with average daily step counts dropping throughout pregnancy despite continued encouragement and walking sessions accompanied by research staff at the biweekly visits. Research from Japan found that daily step counts of 6000 in pregnant people were sufficient to see a significant reduction in blood glucose values [25]. Current Canadian physical activity recommendations for pregnant populations emphasize achieving 150 weekly minutes of physical activity spread over 3 days, without a specific step count recommendation [26]. Thus, the goal of 10,000 daily steps may not be realistic or necessary for pregnant females to achieve GWG goals.

The results of this study support the feasibility of quantitatively evaluating intervention adherence using adherence scoring in a relatively healthy and homogenous pregnancy cohort. The adherence tool has not been validated in an external population. Creation of an adherence score to determine compliance with the specific components of both a diet and exercise intervention will be useful for future research and possibly clinical application. The overall adherence score indicated that intervention participants were able to change their behaviors to be in line with the intervention; however, they did not maintain this change at the same level from middle to late pregnancy. Intervention adherence decreased, with the average intervention participant achieving <50% adherence (adherence score < 2 of a possible 4). Although there was a relatively consistent proportion of participants who maintained their protein (47.3% at 26–28 weeks’ gestation; 40% at 36–38 weeks’ gestation) and dairy protein intake (47.3% at 26–28 weeks’ gestation; 41.8% at 36–38 weeks gestation) through to the end of pregnancy, fewer participants were adherent to the individualized requirement for energy intake and the daily step count portion of the intervention. Although 43.6% of the intervention participants were already meeting their individualized energy intake goals at baseline, this dropped to only 20% by the end of pregnancy. The observation of lower energy intakes than the individualized EERs may reflect underreporting of dietary intake. Alternatively, the estimated EERs may be excessive to meet individual needs because a recent review of energy requirements in pregnancy indicated that pregnant individuals in overweight or obese BMI categories may not require a higher energy intake during pregnancy to achieve GWG recommendations [27]. Recent recommendations from the National Academies of Science, Engineering, and Medicine echo this sentiment and suggest new EER formulas be created to reflect prepregnancy BMI [28]. It should also be noted that energy balance may have been underestimated because the SenseWear v.52 algorithm for energy expenditure compared with the measures of indirect calorimetry is reported to overestimate energy expenditure in pregnant females [29], although, in this study, any bias in this regard would be random and not systematically affect the data on adherence scores.

Lack of adherence to dietary recommendations during pregnancy has been highlighted as a concern but primarily from observational research. An Australian prospective cohort study assessed compliance with dietary recommendations in the Australian Guide to Healthy Eating in pregnancy and postpartum using a FFQ [30]. Ultimately, no females met all Australian Guide to Healthy Eating food group recommendations [30]. Similarly, a birth cohort in New Zealand found that pregnant females were unsuccessful in meeting national dietary guidelines, with 25% of females failing to meet any recommendations for any food group [31]. A Canadian prospective cohort study found that approximately half of the pregnant females classified as normal weight or underweight met Canada’s Food Guide recommendations (54% and 50%), but fewer overweight (47%) or obese (41%) females met the recommendations [32]. The Canadian recommendation for pregnant persons to choose reduced-fat milk was achieved by 56% of the females, whereas <1% of the females consumed the recommended extra 2–3 servings daily from any food group [32].

Although not yet widely used, intervention trials in nonpregnant and pregnant persons are beginning to use scoring systems to quantify and report adherence for defined diet protocols. Mediterranean diet intervention adherence can be assessed using the MedDietScore, whereas the Dixon-DASH dietary index is a tool for quantifying adherences to the Dietary Approaches to Stop Hypertension (DASH) diet [[33], [34], [35]]. Two previous studies on pregnant populations have created their own unique adherence scoring mechanisms. The Fit for Delivery study, a randomized controlled intervention trial aimed at preventing excessive GWG through dietary counseling, constructed a unique intervention adherence score [13]. Dietary adherence was assessed using a 43-question FFQ developed for the Fit for Delivery study, resulting in a score from 1 to 10; higher adherence was reflected by increasing scores [13]. The Nutrition and Exercise Lifestyle Intervention Program (NELIP) study, a lifestyle intervention in pregnant Canadian females designed to lower the incidence of excess GWG, also used a scored adherence system, based on measures such as diet records and attendance at study visits [14]. The score generated consisted of 3 dietary components and 3 exercise components, resulting in a score of 6 [14]. Ultimately, the NELIP study found that participants who gained appropriate amounts of gestational weight had significantly higher intervention adherence scores across all pBMI categories [14].

A nested qualitative study within the BHIP study with control participants found that maintaining healthy practices in pregnancy was easier if they had established such behaviors before pregnancy [36]. This suggests that changes in health behaviors may have felt like an extreme shift for some participants if they were not already making such efforts, making it difficult to maintain these changes until the end of pregnancy. In addition, a common sentiment echoed by pregnant individuals attempting to adapt their health behaviors is that despite initial motivation or desire, other commitments such as childcare and work responsibilities make it more difficult [36,37]. This also raises the question as to whether certain behaviors or characteristics may predict participant adherence and whether interventions can be modified to best address such characteristics. A systematic review of adherence to dietary guidelines in pregnancy in observational studies found maternal education status, smoking status, and maternal age play a role in predicting general dietary guideline adherence throughout pregnancy [38]. Taken together, this suggests that different communication approaches should potentially be taken for different research subpopulations to best promote adherence. However, future research should continue to explore factors beyond demographics, which may promote or restrict adherence within study populations, to better understand and address potential barriers or facilitators of adherence. Moreover, although establishing adherence measurement guidelines is important, what reflects adequate adherence, or what level of adherence is required to generate a clinical difference, must still be determined. In studies in nonpregnant individuals, pharmaceutical interventions and some behavior change interventions have observed 70%–80% adherence to measurements such as fitness class attendance or number of doses consumed [[39], [40], [41], [42], [43]]. One study in pregnancy suggested 70% adherence to a lifestyle intervention reduced excessive GWG [14]. Clearly, more investigation of parameters of compliance with lifestyle interventions in pregnancy is required to ensure success that influences positive clinical outcomes.

Reporting measures of intervention adherence is important to transparency in research and to understand the effectiveness of a trial [44,45]. Our study demonstrated that creating a quantifiable measure of intervention adherence is entirely plausible and can likely be achieved within other interventions. However, there are some important considerations to make when measuring adherence. It is a key to define the specific elements of the intervention before describing adherence; this may be achieved by considering an intervention as a series of specified steps that must be completed [46]. In this study, we considered the main behaviors we asked our participants to define the intervention and, thus, developed 4 specific criteria. Next, one must ensure the proper measurements or tools are available to measure what was delivered and received, as opposed to the intended intervention effect [46]. For example, to measure adherence in our study, we had to determine average daily protein intake. We were able to report on this measure because we had collected data on daily food intake through 3DDR and had access to NutritionistPro software to complete the nutritional analysis, such as macronutrient intake. Without these tools, reporting on protein adherence may not have been possible. Taken together, the foregoing considerations allow reporting intervention adherence to be more achievable.

The key strength of our BHIP study is that we planned a priori to assess adherence to the intervention using 2 measures—one diet quality using the PrimeScreen diet scores and the other an intervention adherence score specifically developed for the BHIP study. The creation and use of a unique adherence score based on objective measures of diet and exercise has rarely been achieved in other RCTs, particularly those in pregnant populations. In this study, accelerometer data were used as an objective measure of daily step count. Although accelerometry has been used in other reported studies in pregnancy [29,47], one study that measured energy expenditure by SenseWear Armband accelerometry, compared with that by indirect calorimetry [48], observed that energy expenditure was overestimated by accelerometry and concluded that pregnancy-specific algorithms should be developed. Thus, energy expenditure by accelerometry could have been overestimated in the BHIP study. Another consideration is that the accelerometers cannot be worn in water, thus not capturing any swimming activity, which is reported as a common exercise undertaken by pregnant individuals [47,48]. In the BHIP study, swimming was unlikely to affect the overall energy expenditure because our detailed records of types of physical activity undertaken showed that only ∼12% of the participants included swimming in the adherence assessment recorded, and this averaged to <4 min/d during the 3 days accelerometry was measured.

For dietary adherence, our measures were more subjective, although we did use high-quality measurement tools, such as 3DDR analyzed by NutritionistPro software that included brand names and weighed measures. Moreover, dietary intake as captured by the 3DDR lowered risk of participant recall bias [49].

This study is not without other limitations. The relatively homogeneous demographics of the study sample may limit the generalizability of our findings to the general pregnant population in Canada. Most participants in both the intervention and control groups were Caucasian, had a secondary education, and reported a high annual household income (> $75,000). Thus, the results might not be replicated in pregnant populations with different sociodemographic characteristics and may not reflect diet and exercise capabilities that are feasible for all pregnant populations. Finally, complete data were required to compute the adherence score but this was limited to 111 (55%) of the participants analyzed for the primary outcome who had complete data collections for all measured parameters necessary for the method assessment. However, the demographic characteristics (Supplementary Table 1) and the mean intakes of protein and energy, energy expenditure and step counts were very similar within and between treatment groups in this analysis and to these measures in the previous published article of the primary outcome of GWG [16].

In summary, the effect of diet and exercise lifestyle interventions in pregnancy may be better understood if behavior change and adherence to treatments measured are reported alongside clinical and metabolic outcomes. If a standard method does not exist to measure adherence to a specific intervention, using a combination of subjective and objective parameters can be applied to report on adherence. In this study, the creation and application of a novel adherence scoring algorithm applied to intervention participants in a diet and exercise lifestyle intervention in pregnancy highlighted low levels of treatment compliance throughout the duration of pregnancy. Low adherence levels may contribute to null or conflicting results seen in research. Future research should aim to explore the factors which facilitate or prevent adherence and how barriers can be overcome.

## Funding

This work was supported by competitive grants from Canadian Institutes of Health Research (CIHR) (FRN123347); Dairy Cluster by Dairy Farmers of Canada and Agriculture and Agri-food Canada (AAFC); and in-kind provision of milk, yogurt, and cottage cheese by GayLea Foods Coop & Ultima Foods, Canada. The funders had no role in the study design, data collection or analysis, decision to publish, or preparation of the manuscript.

## Author disclosures

KD, MFM, and SAA declare that they have no competing interests.

## Data Availability

The data sets used and analyzed for this manuscript can be made available in the future from the corresponding author on reasonable request.

## Declaration of interests

☒ The authors declare the following financial interests/personal relationships which may be considered as potential competing interests: Stephanie Atkinson reports financial support was provided by Canadian Institutes of Health Research. Stephanie Atkinson reports financial support was provided by Dairy Cluster by Dairy Farmers of Canada, and Agriculture and Agri-food Canada (AAFC). Stephanie Atkinson reports equipment, drugs, or supplies was provided by GayLea Foods Coop & Ultima Foods, Canada.

## Appendix A Supplementary data

Multimedia component1 Multimedia component1

## References

[1] McGiveron A., Foster S., Pearce J., Taylor M.A., McMullen S., Langley-Evans S.C. (2015). Limiting antenatal weight gain improves maternal health outcomes in severely obese pregnant women: findings of a pragmatic evaluation of a midwife-led intervention. J. Hum. Nutr. Diet..

[2] Ferreira D.L.S., Williams D.M., Kangas A.J., Soininen P., Ala-Korpela M., Smith G.D. (2017). Association of pre-pregnancy body mass index with offspring metabolic profile: analyses of 3 European prospective birth cohorts. PLOS Med.

[3] Rooney B.L., Mathiason M.A., Schauberger C.W. (2011). Predictors of obesity in childhood, adolescence, and adulthood in a birth cohort. Matern. Child Health J..

[4] Mamun A.A., Mannan M., Doi S.A.R. (2014). Gestational weight gain in relation to offspring obesity over the life course: a systematic review and bias-adjusted meta-analysis. Obes. Rev..

[5] Shin D., Song W.O. (2015). Prepregnancy body mass index is an independent risk factor for gestational hypertension, gestational diabetes, preterm labor, and small- and large-for-gestational-age infants. J. Matern. Fetal Neonatal Med..

[6] Institute of Medicine, National Research Council (2009).

[7] McDonald S.D., Woolcott C., Chapinal N., Guo Y., Murphy P., Dzakpasu S. (2018). Interprovincial variation in pre-pregnancy body mass index and gestational weight gain and their impact on neonatal birth weight with respect to small and large for gestational age. Can. J. Public Health..

[8] Gardner B., Wardle J., Poston L., Croker H. (2011). Changing diet and physical activity to reduce gestational weight gain: a meta-analysis. Obes. Rev..

[9] Teede H.J., Bailey C., Moran L.J., Bahri Khomami M.B., Enticott J., Ranasinha S. (2022). Association of antenatal diet and physical activity-based interventions with gestational weight gain and pregnant outcomes: a systematic review and meta-analysis. JAMA Intern. Med..

[10] Muktabhant B., Lawrie T.A., Lumbiganon P., Laopaiboon M. (2015). Diet or exercise, or both, for preventing excessive weight gain in pregnancy. Cochrane Database Syst. Rev..

[11] Asbee S.M., Jenkins T.R., Butler J.R., White J., Elliot M., Rutledge A. (2009). Preventing excessive weight gain during pregnancy through dietary and lifestyle counseling: a randomized controlled trial. Obstet. Gynecol..

[12] Oostdam N., Van Poppel M.N.M., Wouters M.G.A.J., Eekhoff E.M.W., Bekedam D.J., Kuchenbecker W.K.H. (2012). No effect of the FitFor2 exercise programme on blood glucose, insulin sensitivity, and birthweight in pregnant women who were overweight and at risk for gestational diabetes: results of a randomised controlled trial. B.J.O.G..

[13] Øverby N.C., Hillesund E.R., Sagedal L.R., Vistad I., Bere E. (2015). The Fit for Delivery study: rationale for the recommendations and test-retest reliability of a dietary score measuring adherence to 10 specific recommendations for prevention of excessive weight gain during pregnancy. Matern. Child Nutr..

[14] Nagpal T.S., Prapavessis H., Campbell C., Mottola M.F. (2017). Measuring adherence to a nutrition and exercise lifestyle intervention: is program adherence related to excessive gestational weight gain?. Behav. Anal. Pract..

[15] Perreault M., Atkinson S.A., Mottola M.F., Phillips S.M., Bracken K., Hutton E.K. (2018). Structured diet and exercise guidance in pregnancy to improve health in women and their offspring: study protocol for the Be Healthy in Pregnancy (BHIP) randomized controlled trial. Trials.

[16] Atkinson S.A., Maran A., Dempsey K., Perreault M., Vanniyasingam T., Phillips S.M. (2022). Be Healthy in Pregnancy (BHIP): a randomized controlled trial of nutrition and exercise intervention from early pregnancy to achieve recommended gestational weight gain. Nutrients.

[17] Health Canada (2010). https://publications.gc.ca/collections/collection_2011/sc-hc/H164-126-2010-eng.pdf.

[18] Health Canada (2018). https://health.canada.ca/en/health-canada/services/food-nutrition/healthy-eating/prenatal-nutrition/pregnancy-weight-gain-calculator.html.

[19] Institute of Medicine (2005). https://nap.nationalacademies.org/catalog/10490/dietary-reference-intakes-for-energy-carbohydrate-fiber-fat-fatty-acids-cholesterol-protein-and-amino-acids.

[20] Rifas-Shiman S.L., Willett W.C., Lobb R., Kotch J., Dart C., Gillman M.W. (2001). PrimeScreen, a brief dietary screening tool: reproducibility and comparability with both a longer food frequency questionnaire and biomarkers. Public Health Nutr.

[21] Szwajcer E.M., Hiddink G.J., Koelen M.A., van Woerkum C.M.J. (2007). Nutrition awareness and pregnancy: implications for the life course perspective. Eur. J. Obstet. Gynecol. Reprod. Biol..

[22] Fowles E.R., Fowles S.L. (2008). Healthy eating during pregnancy: determinants and supportive strategies. J. Community Health Nurs..

[23] Phelan S. (2010). Pregnancy: a “teachable moment” for weight control and obesity prevention. Am. J. Obstet. Gynecol..

[24] Forbes L.E., Graham J.E., Berglund C., Bell R.C. (2018). Dietary change during pregnancy and women’s reasons for change. Nutrients.

[25] Hayashi A., Oguchi H., Kozawa Y., Ban Y., Shinoda J., Suganuma N. (2018). Daily walking is effective for the management of pregnant women with gestational diabetes mellitus. J. Obstet. Gynaecol. Res..

[26] Mottola M.F., Davenport M.H., Ruchat S.M., Davies G.A., Poitras V., Gray C. (2018). No. 367-2019 Canadian guideline for physical activity throughout pregnancy-No. 367-2019. J. Obstet. Gynaecol. Can..

[27] Most J., Dervis S., Haman F., Adamo K.B., Redman L.M. (2019). Energy intake requirements in pregnancy. Nutrients.

[28] National Academies of Sciences (2020). Proceedings of the a Workshop.

[29] Most J., Vallo P.M., Gilmore L.A., St Amant M.St., Hsia D.S., Altazan A.D. (2018). Energy expenditure in pregnant women with obesity does not support energy intake recommendations. Obesity (Silver Spring).

[30] Blumfield M.L., Hure A.J., MacDonald-Wicks L.K., Patterson A.J., Smith R., Collins C.E. (2011). Disparities exist between National food group recommendations and the dietary intakes of women. B.M.C. Womens Health..

[31] Morton S.M.B., Grant C.C., Wall C.R., Atatoan Carr P.E.A., Bandara D.K., Schmidt J.M. (2014). Adherence to nutritional guidelines in pregnancy: evidence from the Growing UP in New Zealand birth cohort study. Public Health Nutr.

[32] Jarman M., Bell R.C., Nerenberg K., Robson P.J. (2017). Adherence to Canada’s Food Guide recommendations during pregnancy: Nutritional epidemiology and public health. Curr. Dev. Nutr..

[33] Panagiotakos D.B., Pitsavos C., Arvaniti F., Stefanadis C. (2007). Adherence to the Mediterranean food pattern predicts the prevalence of hypertension, hypercholesterolemia, diabetes and obesity, among healthy adults; the accuracy of the MedDietScore. Prev. Med..

[34] USDA/DHHS (2005). https://health.gov/sites/default/files/2020-01/DGA2005.pdf.

[35] Dixon L.B., Subar A.F., Peters U., Weissfeld J.L., Bresalier R.S., Risch A. (2007). Adherence to the USDA food guide, DASH eating plan, and Mediterranean dietary pattern reduces risk of colorectal adenoma. J. Nutr..

[36] Murray-Davis B., Grenier L., Atkinson S.A., Mottola M.F., Wahoush O., Thabane L. (2019). Experiences regarding nutrition and exercise among women during early postpartum: a qualitative grounded theory study. B.M.C. Pregnancy Childbirth..

[37] McKinley M.C., Allen-Walker V., McGirr C., Rooney C., Woodside J.V. (2018). Weight loss after pregnancy: challenges and opportunities. Nutr. Res. Rev..

[38] Caut C., Leach M., Steel A. (2020). Dietary guideline adherence during preconception and pregnancy: a systematic review. Matern. Child Nutr..

[39] DiMatteo M.R. (2004). Variations in patients’ adherence to medical recommendations: a quantitative review of 50 years of research. Med. Care.

[40] Fischer M.A., Stedman M.R., Lii J., Vogeli C., Shrank W.H., Brookhart M.A. (2010). Primary medication non-adherence: analysis of 195,930 electronic prescriptions. J. Gen. Intern. Med..

[41] Koehorst-ter Huurne K., Movig K., Van Der Valk P., Van Der Palen J., Brusse-Keizer M. (2015). Differences in adherence to common inhaled medications in COPD. C.O.P.D..

[42] Haakstad L.A.H., Bø K. (2011). Exercise in pregnant women and birth weight: a randomized controlled trial. B.M.C. Pregnancy Childbirth..

[43] Domingues M.R., Bassani D.G., da Silva S.G., Coll C., da Silva B.G.C., Hallal P.C. (2015). Physical activity during pregnancy and maternal-child health (PAMELA): study protocol for a randomized controlled trial. Trials.

[44] Craig P., Dieppe P., Macintyre S., Michie S., Nazareth I., Petticrew M. (2008). Developing and evaluating complex interventions: the new Medical Research Council guidance. BMJ.

[45] Hawe P., Shiell A., Riley T. (2004). Complex interventions: how “out of control” can a randomised controlled trial be?. BMJ.

[46] Graham L., Wright J., Walwyn R., Russell A.M., Bryant L., Farrin A. (2016). Measurement of adherence in a randomised controlled trial of a complex intervention: supported self-management for adults with learning disability and type 2 diabetes. B.M.C. Med. Res. Methodol..

[47] Nascimento S.L., Surita F.G., Godoy A.C., Kasawara K.T., Morais S.S. (2015). Physical activity patterns and factors related to exercise during pregnancy: a cross sectional study. PLOS ONE.

[48] Smith K.M., Lanningham-Foster L.M., Welk G.J., Campbell C.G. (2012). Validity of the SenseWear® Armband to predict energy expenditure in pregnant women. Med. Sci. Sports Exerc..

[49] Crawford P.B., Obarzanek E., Morrison J., Sabry Z.I. (1994). Comparative advantage of 3-day food records over 24-hour recall and 5-day food frequency validated by observation of 9- and 10-year-old girls. J. Am. Diet. Assoc..

