# Supplementary material for: Comparative Assessment of Diet Quality and Adherence to a Structured Nutrition and Exercise Intervention Compared with Usual Care in Pregnancy in a Randomized Trial
Source: Curr Dev Nutr. 2023 May 13;7(6):100097. doi: 10.1016/j.cdnut.2023.100097 (PMC10334218; doi:10.1016/j.cdnut.2023.100097)
Supplement: Multimedia component1 [file mmc1.pdf]

Dempsey K. Comparative assessment of diet quality and adherence to a structured nutrition and exercise intervention versus usual care in pregnancy in a randomized trial.

Supplementary Table 1: Baseline (12-17 weeks gestation) demographic characteristics of participants included in analysis of diet quality and adherence in the Be Healthy in Pregnancy study (N = 111).

**Characteristic**	**Descriptive statistics**	
	**Intervention (N = 55)**	**Control (N = 56)**
Age at enrolment (years)	30.9±3.64[1]	31.8±3.89
pBMI (kg/m^2)	25.1±4.1	24.7±4.7
pBMI category		
Underweight (< 18.5)	0 (0.0)[2]	1 (1.8)
Normal Weight (18.5-24.9)	34 (61.8)	34 (60.7)
Overweight (25.0-29.9)	15 (27.3)	13 (23.2)
Obese (> 30.0)	6 (10.9)	8 (14.3)
Education Level		
Secondary school	1 (1.8)	1 (1.8)
Post-secondary school or greater	54 (98.2)	55 (98.2)
Total annual household income		
< $45,000	2 (3.6)	5 (8.9)
$45,000-$75,000	10 (18.2)	6 (10.7)
≥ $75,000	42 (76.4)	40 (71.4)
No response	1 (1.8)	5 (8.9)
Marital Status		
Married/living with partner	55 (100.0)	53 (94.6)
Ethnicity		
Caucasian	48 (87.3)	49 (87.5)
Other	7 (12.7)	7 (12.5)
Parity		

Nulliparous	28 (50.9)	26 (46.4)
Primip/Multiparous	27 (49.1)	30 (53.6)

[1] Mean±SD[2] n(%) for categorical variables
